# Region-Resolved Integrative Multi-Omic Characterization Reveals Diverse Tumor and Microenvironment Features of Pituitary Neuroendocrine Tumors

**DOI:** 10.1016/j.mcpro.2026.101583

**Published:** 2026-05-12

**Authors:** Tao Xie, Qiang Xie, Qin Hu, Jiamin Hu, Yang Gao, Rongkui Luo, Chaolong Yan, Pin Chen, Zijiang Yang, Yi Liu, George Takura Tabengwa, Xiaobiao Zhang

**Affiliations:** 1 Department of Neurosurgery, Zhongshan Hospital, Fudan University, Shanghai, China; 2 Cancer Center, Shanghai Zhongshan Hospital, Fudan University, Shanghai, China; 3 Department of Pathology, Zhongshan Hospital, Fudan University, Shanghai, China; 4 Digital Medical Research Center, Fudan University, Shanghai, China

**Keywords:** PitNETs, cavernous sinus region, saddle region, multi-omics research, CAFs

## Abstract

Pituitary neuroendocrine tumors are frequently invasive, with cavernous sinus invasion leading to poor treatment outcomes and high recurrence. Regional differences within these tumors remain poorly understood, hindering targeted therapy development. Here, we present the first integrative multi-omics analysis combining proteomics, metabolomics and single-cell transcriptomics to characterize tumors from the cavernous sinus and saddle regions. Our results reveal profound regional and cellular heterogeneity: cavernous sinus tumors exhibit significantly enhanced cell proliferation, driven by cancer-associated fibroblasts through the IGF1-IGF1R-MAPK1 axis. Cancer-associated fibroblasts in the cavernous sinus secrete IGF1 under regulation of the transcription factor FOXO1, which binds to receptors on tumor cells to activate proliferation. Metabolomic profiling identifies proline as a key enriched metabolite that stimulates cancer-associated fibroblasts to produce collagen fibers, reinforcing a pro-tumorigenic microenvironment. Single-cell transcriptomics further delineates a distinct subpopulation of receptor-positive malignant cells and a high abundance of cancer-associated fibroblasts in the cavernous sinus. These findings establish core mechanisms underlying the aggressive behavior of cavernous sinus-invading tumors, providing novel actionable targets for precision therapeutic strategies tailored to distinct tumor regions.

## Highlight

• Multi-omics reveals cavernous sinus vs. saddle regional differences in PitNETs.
• CAFs and IGF1R+ tumor cells drive cavernous sinus tumor aggression.
• Cross-talk between tumor cells and CAFs promotes proliferation.
• CAFs-tumor cell crosstalk relies mainly on IGF signaling and proline metabolism.

## Graphical Abstract

**Figure:**
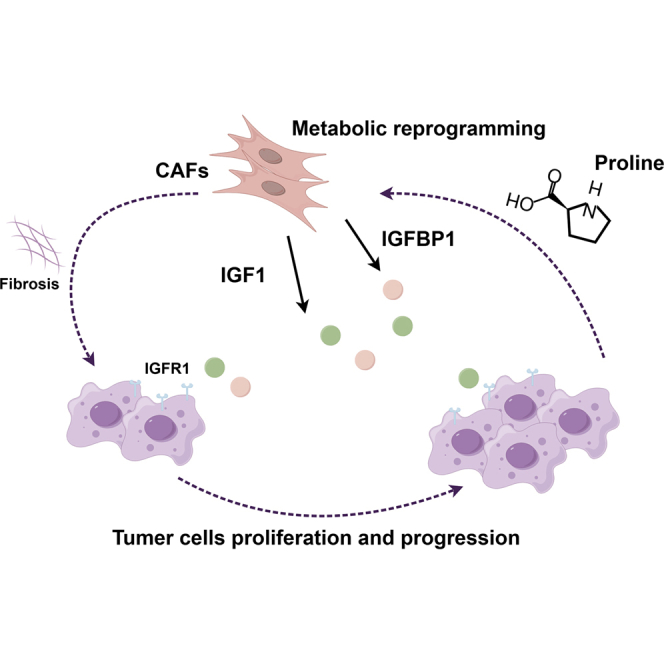

## In Brief

This study presents the first region-resolved, multi-omic characterization of pituitary neuroendocrine tumors (PitNETs), revealing distinct molecular and microenvironmental features between cavernous sinus–invasive and saddle-localized tumors. By integrating single-cell transcriptomics, proteomics, and metabolomics, we uncover a critical role for IGF1R signaling and cancer-associated fibroblasts in driving regional aggressiveness—providing new insights into PitNET heterogeneity and potential therapeutic targets.

Pituitary neuroendocrine tumors (PitNETs) are tumors originating from the anterior pituitary gland. The incidence rate of PitNETs is between 3.9 and 7.4 cases per 100,000 per year (1). In the WHO fifth edition classification of pituitary tumors, the terminology was changed from "pituitary adenoma" to "pituitary neuroendocrine tumor" to better align with terminology for other neuroendocrine neoplasms (2). Around 35% to 65% of PitNETs demonstrate invasion into surrounding structures such as the sphenoid sinus, cavernous sinus, and suprasellar cistern (3). These invasive PitNETs are associated with lower rates of gross total resection, higher surgical complications, and increased risk of recurrence and progression compared to non-invasive PitNETs (4, 5, 6). We know little about the biological characteristics of invasive PitNETs. Previously, we knew the invasive biological characteristics of PitNETs through intertumor heterogeneity; little research focuses on the intratumor heterogeneity, which may limit our treatment options.

The main therapies for PitNETs include surgery, medication, and radiation, with surgery being the primary treatment (7, 8, 9, 10). However, one surgical difficulty is managing tumors invading the cavernous sinus, which is difficult to remove. With the advancement of endoscopic transsphenoidal techniques and the progress of cavernous sinus (CS) anatomy (11), fully exposure of CS spaces and suction of the soft tumors during the neurovascular space is possible (12, 13). However, in clinical practice, we often found tumor in the CS was stiffer than tumor in the saddle and could only be partially resected (12). This suggests significant biological differences exist between the saddle and the cavernous components.

Intratumor heterogeneity is an emerging hallmark of cancer. Next-generation sequencing and single-cell sequencing technologies have enabled scientists to uncover substantial diversity between cells within the same tumor. Studies have revealed intratumor heterogeneity in various cancer types at the genetic, epigenetic, and phenotypic levels. Perhaps most striking is the phenotypic diversity uncovered between cancer cell subpopulations within a single lesion. Distinct differentiation states and drug sensitivities have been documented, suggesting intratumor evolution has generated multiple subclones adapted to different microenvironments or therapeutic pressures. This heterogeneity poses challenges for cancer treatment and limits the efficacy of conventional therapies designed to target the average tumor profile.

Previously, the focus of studies has not been on the disparities between the saddle and cavernous regions of invasive PitNETs. However, in our pioneering effort, we have taken a groundbreaking step to bridge this knowledge gap. For the first time, we have employed multi-omics techniques to delve into the distinct spatial zones of these tumors and unveil the heterogeneity within. Our objective is to unearth potential prognostic indicators and therapeutic targets that have hitherto remained undiscovered due to the averaging of heterogeneous tumor profiles in previous investigations. By doing so, we hope to shed new light on the understanding and treatment of these tumors.

## Experimental procedures

### Sample Collection and Extraction

We enrolled 10 patients diagnosed with Knosp grade 4 PitNETs in our study. For each patient, specimens were obtained from both the cavernous sinus and saddle regions. Pituitary tissues were obtained post-mortem from donors who had deceased due to unrelated causes, with no documented history of pituitary-related disorders in their medical records. All tissue acquisitions were conducted in accordance with strict ethical guidelines and were approved by the institutional review board. The collection of these specimens was carried out in accordance with standard clinical procedures. Based on the technical guidance provided by the company, samples were separately extracted for proteomics, metabolomics, and single-cell transcriptomics analyses to comprehensively characterize the molecular and cellular landscapes of the PitNETs.

### Biospecimen Processing in Accordance With Clinical Proteomics Checklist

Biospecimen processing methods were performed in accordance with Section 4 (Biospecimen Qualifications) of the clinical proteomics checklist. Tissue specimens (average weight: 45 mg) were snap-frozen in liquid nitrogen immediately after surgical resection at Zhongshan Hospital and stored at −80 °C until further processing. For proteomic analysis, frozen tissues were lysed using a Urea lysis buffer (8 M urea, 100 mM Tris-HCl, pH 8.0) for protein extraction. All snap-frozen tissue specimens used in proteomics experiments underwent standard histological review by a qualified pathologist to confirm the diagnosis, assess the percentage of tumor cellularity, and evaluate the viability of tissue sections, ensuring the quality and relevance of the analyzed biospecimens. Specifically, the adjacent segment of tumor tissue was formalin-fixed paraffin-embedded (FFPE) and stained with hematoxylin and eosin (H&E) at Zhongshan Hospital for quality assessment.

#### Isolating Proteins and Enzymatic Proteolysis

For the isolation of proteins and their subsequent tryptic digestion, approximately 45 mg of frozen, ground PitNET tissues or APGs were individually homogenized in an adequate amount of Urea lysis buffer (8 M urea, 100 mM Tris-HCl, pH 8.0), supplemented with protease inhibitors (provided by Thermo Scientific). The resulting lysate was centrifuged at 4 °C for 15 min at 16,000×*g*. Protein concentrations were determined by the BCA assay. Approximately 4900 μg of proteins were extracted from each specimen. For proteomic analysis, 50 μg of proteins were adjusted to a final concentration of 5 mM with dithiothreitol (DTT) and incubated at 56 °C for 30 min. They were then treated with 20 mM (a final concentration) iodoacetamide (IAA) and incubated at room temperature in the dark, following the FASP protocol (14). After a 30-min incubation, the samples were again adjusted to a final concentration of 5 mM DTT and kept in the dark for an additional 15 min. The protein samples were loaded in 30 kD Microcon centrifugal filters, and centrifuged at 12,000×*g* for 20 min, washed twice with Urea lysis buffer and 50 mM ammonium bicarbonate. The proteins were digested with trypsin overnight at 37 °C, using an enzyme-to-substrate ratio of 1:25. The resulting peptides were dehydrated using a SpeedVac concentrator (Eppendorf).

#### Liquid Chromatography-Tandem MS

Peptide mixtures were evaluated using an Easy-nLC 1200 ultra-high-pressure liquid chromatography (UHPLC) system coupled to a Fusion Lumos mass spectrometer from Thermo Fisher Scientific. The samples were introduced into a self-made pre-column (100 μm × 2 cm; 120 Å pore size, 3 μm C18 particle size; (SunChrom)), followed by separation with a linear gradient from 4% to 100% solvent B (consisting of 80% acetonitrile and 0.1% formic acid) at a flow rate of 600 nl/min over a period of 150 min through a custom-made silica capillary column (150 μm × 30 cm; 120 Å pore size, 1.9 μm C18 particle size; (SunChrom)).

Experiments integrating LC-MS/MS were performed using Field Asymmetric Ion Mobility Spectrometry (FAIMS). Specifically, FAIMS voltage settings were adjusted to −40V, −60V, and −80V, while other parameters remained uniform as described before (15), configured as follows: protein quantification involved an MS1 scan at a resolution of 120,000 (at 200 m/z), with an AGC target of 5e5, maximum injection time of 50 ms, and a scan range of 350 to 1500 m/z. MS2 scans employed higher-energy collision dissociation in the Ion Trap (Ion trap scan rate set to rapid, isolation window of 1.6 m/z, maximum injection time of 10 ms, AGC target of 1e4, and a normalized collision energy of 30%). The dynamic exclusion duration for previously detected precursor ions was set to 45 s, with a cycle time of 1 s.

### MS Data Analysis

#### Peptide and Protein Detection

MS data from the proteomics study were analyzed using "Firmiana" a comprehensive online proteomics platform (16), against the human protein database (27,414 protein sequences) in NCBI's RefSeq repository with Mascot 2.4 as the search engine. A precursor mass tolerance of 20 ppm and a product ion tolerance of 0.5 Da were permitted. The search allowed for a maximum of two missed tryptic cleavages. Carbamidomethylation of cysteines was designated as a fixed modification, while N-terminal acetylation and methionine oxidation were considered as variable modifications. To ensure the accuracy of protein identification, a target-decoy approach was implemented to maintain the false discovery rate (FDR) for peptides and proteins at less than 1%. Percolator was utilized to calculate the q-value and to ensure that the FDR, as determined by decoy hits, for each peptide-spectrum match (PSM) was below 1%. Subsequently, peptides with fewer than seven amino acids were filtered out. The ion score threshold for peptide identification was set at 20. For enhanced quality control, all PSMs across samples were pooled for protein-level FDR assessment. The q-values for both target and decoy peptide sequences were incrementally adjusted until the protein FDR was less than 1%, adhering to the principle of parsimony. Ultimately, proteins identified by at least two distinct peptides were chosen for further analysis to minimize the risk of false positives.

#### MS Quantification of Proteins

Proteomic data were analyzed with Firmiana, integrating both output and raw mzXML file data (16).

Identified peptides were extracted. To generate extracted-ion chromatograms (XICs), MS1 identification details were used. Peptide abundance was quantified as the area under the XIC curve. A non-redundant list of peptides was used to infer proteins according to the principle of parsimony. Protein abundance was estimated using a label-free, intensity-based absolute quantification (iBAQ) method. Particularly, iBAQ calculates the ratio of the sum of identified peptide intensities to the number of theoretically observable peptides per protein (17). The Fraction of Total (FOT), a relative quantification metric, was derived by normalizing a protein’s iBAQ value against the total iBAQ of all identified proteins in the study. For clarity of presentation, FOT values were scaled by a factor of 1 × 10^5^ (15).

#### Missing Value Imputation

Proteins exhibiting a missing rate below 50% were individually estimated using data stratified by clinicopathological classification, aligning with prior studies. The imputation of missing values was conducted through the K-nearest neighbor (KNN) method, leveraging the "impute" R package (RRID:SCR_009245, 10.18129/B9.bioc.impute), with the selection of the five closest neighbors for each calculation.

#### MS Data Validation

Peptides derived from the digestion of HEK293T cells (Cat# CRL-11268, RRID: CVCL_QW54), sourced from the National Infrastructure Cell Line Resource, were subjected to LC-MS/MS analysis to assess instrument performance at 4-run intervals. The digestion and analysis of HEK293T cells adhered to the same protocol and parameters as those used for PitNET samples. Spearman’s rank correlation coefficients were calculated for all quality control runs using R version 4.0.2. The average correlation coefficient among quality control runs was 0.92.

### Protein Expression-Based Cluster Analysis

To understand the protein abundance relationships between tumors, we performed hierarchical clustering on variable genes. The protein expression matrix of the 20 tumor samples was used to identify the proteomic clusters using the consensus cluster method. Consensus clustering was performed using the ConsensusClusterPlus (R package, v.1.48.0), using the top 50% most variable proteins. The following detail settings were used for clustering: number of repetitions = 1000 bootstraps; pItem = 0.8 (resampling 80% of any sample); pFeature = 1 (resampling 100% of any protein); clusterAlg = “pam”; and distance = “spearman”.

The consensus matrices for k = 2, 3, 4, and 5 clusters are shown in Supplemental Fig. S1. Subtype clusters were selected based on the change in the area under the cumulative distribution function curve, with the number of clusters selected being the minimum value that would capture most of the information. We selected a 2-cluster solution as the best for the consensus matrix, with k = 2 deemed to yield the cleanest separation among clusters, and the consensus CDF and delta plot showed little increase in area for k = 2 compared to k = 3.

### Pathway Enrichment Analysis

To unravel the biological mechanisms linked to our dataset, we conducted enrichment analyses of pathways utilizing Metascape (https://metascape.org/gp/index.html#/main/step1) and ConsensusPathwayDB (http://cpdb.molgen.mpg.de/). The significance of the pathways identified was determined using Fisher's exact test, which was based on KEGG pathways and a range of categorical annotations. These annotations included the Gene Ontology (GO RRID: SCR_002811) term for 'biological process' and Reactome, offering a broad perspective on the disrupted biological networks within our study.

### Functional Enrichment Analysis of Proteomics Data With GSVA

To probe the biological characteristics of our heterogeneous sample set more thoroughly, we utilized single-sample gene set enrichment analysis (ssGSEA/GSVA) (RRID:SCR_021058). This method harnessed the gene expression patterns from the proteomic data across different samples to calculate enrichment scores for ontological gene sets. The analysis was conducted with the GSVA package (18, 19) available in R/Bioconductor. For evaluating the statistical significance of the pathway enrichment scores (PES) across samples, we employed a linear model adjusted with the F-statistic, facilitated by the limma package (RRID: SCR_010943) (20). in R/Bioconductor. The PES significance was then corrected using the Benjamini–Hochberg procedure, applying a stringent threshold of an adjusted *p*-value of 0.05, to ensure the validity and dependability of our results.

### Multi-Gene Proliferation Scores (MGPS)

For MPGS analysis, following previously published work (21), MGPS was the mean expression of gene-wise z-scores for protein expression for all cell cycle-regulated genes identified by Whitfield *et al*. in each sample (22, 23).

### Construction of Protein–Protein Interaction Network

To elucidate the intricate web of interactions among proteins, we harnessed the power of STRING v11.0 (RRID:SCR_005223, https://string-db.org/). Employing a medium confidence threshold of 0.4, we incorporated both experimental data and database information as our primary sources for active interactions. The resulting intricate network was brought to life through the visualization capabilities of Cytoscape version 3.5.196, providing a comprehensive view of the protein landscape and its interplay within the cellular context.

### The Extraction of Lipids and Metabolites

The sample was thawing slowly at 4 °C, and 30 mg of tissue was transferred to a 1.5 ml centrifuge tube. A pre-cooled methanol/water solution (2:1) of 300 μl was added to the tube, followed by vortexing for 30 s to homogenize the tissue. Next, 600 μl of MTBE solution was added, and the tube was vortexed again for 30 s. The tube was then cooled in an ice bath and subjected to ultrasonication for 10 min. After ultrasonication, the tube was centrifuged at 14,000 g and 4 °C for 10 min, resulting in the separation of the solution into upper and lower layers. The upper layer, containing the lipids, was collected in a new EP tube, while the lower layer, containing the metabolites, was collected in a different EP tube. Finally, the samples were dried under vacuum at room temperature and stored at −80 °C.

### LC-MS/MS Analysis of Lipids

The mobile phase A and B are identical in both negative and positive modes. Thereinto, mobile phase A was consisted of 10 mM ammonium formate, acetonitrile and water in a 60:40 ratio, along with 0.1% formic acid; mobile phase B consisted of 10 mM ammonium formate, isopropyl alcohol and acetonitrile in a ratio of 50:50, along with 0.1% formic acid. The sample was separated using the microflow rate ultra-high performance liquid chromatography system Nexera UHPLC LC-30A. Initially, the chromatographic column was equilibrated with 98% of mobile phase A. Subsequently, the sample was delivered to the Lipid column (Thermo, Acclaim C30, 3 μm, 2.1 × 100 mm column) using an autosampler at a flow rate of 0.26 ml/min. The gradient elution was performed as follows: starting with 30% of mobile phase B, followed by a linear increase from 30% to 100% of mobile phase B over the next 20 min. After 5 min at 100% of mobile phase B, there was a 0.1-min adjustment back to 30% of mobile phase B, followed by a 2.9-min cleaning step.

After the separation on the chromatographic column, the samples were analyzed using the Q Exactive HF-X mass spectrometer. The detection was performed in both positive and negative ion modes. The full scan range for the parent ions was set from 200 to 2000 m/z. For MS1, the resolution was set to 120,000, with an Automatic Gain Control (AGC) target set to 1e6 and a maximum ionization time (Maximum IT) of 100 ms. For MS2, the resolution was set to 15,000, with an AGC target of 2e5 and a maximum ionization time of 80 ms. The fragmentation was carried out using the High-Energy Collision Dissociation (HCD) mode, with normalized collision energy values of 20, 40, and 60. The isolation window was set at 1.5 m/z.

### Lipid Identification and Quantification

The raw data collected from the mass spectrometry analysis (RAW files) were processed using Progenesis QI software, a commercial lipidomics software platform developed by Waters, for database searching. The Lipid-MAPS was utilized as the target database. The search parameters included a parent tolerance of 5 ppm and a product tolerance of 5 ppm, ultimately obtaining identification information for the samples. We retained peak area data for which missing values did not exceed 50% within the experimental samples, and excluded peak area data points with a relative standard deviation (RSD) exceeding 30% within the quality control (QC) samples. Specifically, missing values were imputed using a method involving one-10th of the minimum value for each respective feature. Finally, annotate the obtained high-quality features according to the LIPID MAPS database.

### LC-MS/MS Analysis of Metabolites

The positive-mode mobile phase A consists of 10 mM ammonium acetate, acetonitrile, and water in a ratio of 95:5, along with 0.1% formic acid. Conversely, the positive mode mobile phase B comprises 10 mM ammonium acetate, acetonitrile, and water in a ratio of 50:50, along with 0.1% formic acid. On the other hand, the negative mode mobile phase A consists of 10 mM ammonium acetate and a mixture of acetonitrile and water in a ratio of 95:5, with the pH adjusted to 8.0 using ammonia solution. Similarly, the negative-mode mobile phase B consists of 10 mM ammonium acetate and a mixture of acetonitrile and water in a ratio of 50:50, with the pH adjusted to 8.0 using ammonia solution.

The sample was analyzed using the Nexera UHPLC LC-30A system. The chromatographic column was initially equilibrated with 98% mobile phase A. The sample was then delivered to the HILIC column (Waters, ACQUITY UPLC BEH Amide 1.7 μm, 2.1 × 100 mm column) via an autosampler at a flow rate of 0.3 ml/min. The gradient elution process involved starting with 2% mobile phase B and maintaining it for 0.5 min. This was followed by a linear increase from 2% to 98% mobile phase B over the next 11.5 min. After maintaining 98% mobile phase B for 4 min, there was a brief adjustment back to 2% mobile phase B for 0.1 min. This was then followed by a 1.9-min cleaning step.

Following separation on the chromatographic column, the samples were analyzed using the Q Exactive HF-X mass spectrometer in both positive and negative ion modes. The parent ions were scanned within a range of 70 to 1050 m/z. In the first stage of mass spectrometry (MS1), the resolution was set to 120,000. An Automatic Gain Control (AGC) target of 3e6 was also set, along with a maximum ionization time of 100 ms. In the second stage (MS2), the resolution was set to 7,500, with an AGC target of 2e5 and a maximum ionization time of 50 ms. The High-Energy Collision Dissociation (HCD) mode was used to fragment the sample, with normalized collision energy values of 20, 40, and 60. The isolation window was set at 1.5 m/z, and the daughter ions were scanned in the range of 200 to 2000 m/z.

For Metabolite identification and quantification, the MS raw files were analyzed by Compound Discoverer (Thermo Fisher Scientific). The chemical standards and manually curated compound list were identified by our in-house metabolite library of mzCloud and mzVault databases based on accurate mass (m/z, ±5 ppm), retention time, and spectral patterns. Next, the identified compounds were also searched against public databases, including the human metabolome database (HMDB) and KEGG (KEGG; RRID: SCR_012773) compound database for further annotation.

### Single-Nuclei RNA Sequencing

#### Preparation and Sequencing of Single-Nuclei RNA Libraries

The single-nuclei RNA library construction followed previously published work (24). Particularly, around 20 to 30 mg of frozen powdered material from pituitary tumor samples was reconstituted in a lysis buffer containing 10 mM Tris-HCl (pH 7.4), 10 mM NaCl, 3 mM MgCl_2_, and 0.1% NP-40. The mixture was gently pipetted 6 to 8 times, placed on ice for 30 s, and then pipetted an additional 4 to 6 times. The resulting lysate, now containing isolated nuclei, was passed through a 40 μm cell strainer. The strainer was rinsed with 1 ml of a buffer solution (1 × PBS with 2% BSA and 0.2 U/μl RNase inhibitor), and the rinse was combined with the initial filtrate. After centrifugation at 500×*g* for 6 min at 4 °C, the nuclear pellet was resuspended in 500 μl of the same buffer. Following DRAQ5 staining, nuclei were refined through fluorescence-activated cell sorting (FACS). The FACS-refined nuclei were centrifuged, resuspended in a minimal volume (approximately 30 μl), and then counted and inspected under a microscope for quality. The nuclei were then diluted to a concentration of about 1000 nuclei/μl, and approximately 20,000 nuclei were prepared for single-nuclei RNA sequencing (snRNA-seq) using the 10 × Chromium platform. Nuclei were loaded onto a Chromium Chip B Single Cell Kit, 48 rxns, and processed through a Chromium Controller to form GEMs. Sequencing library preparation was carried out with the Chromium Single Cell 3′ GEM, Library & Gel Bead Kit v3, 16 rxns, following the manufacturer's instructions. The sequencing was conducted on an Illumina NovaSeq 6000 S4 flow cell, using the XP workflow with a 28 × 8 × 98 bp recipe.

#### Preprocessing of Single-Nuclei RNA Sequencing Data

We processed the raw data from each sample by running the demultiplexed FASTQ files through the Cell Ranger v3.1.0 software using its 'count' command with standard settings, and we also created a custom pre-mRNA GRCh38 reference genome to include both exonic and intronic reads. This custom genome reference updated the transcript annotations from the 10× Genomics standard human genome reference 3.0.0 (GRCh38 and Ensembl 93).

For further analysis, we employed Seurat v3.1.2 (RRID:SCR_007322). A Seurat object was established for each sample using the initial feature-barcode matrix. We applied a suite of quality control filters to eliminate cell barcodes that met any of the criteria suggested by Seurat: low total transcript counts (fewer than 300); potential debris with minimal gene expression (fewer than 200) and low UMIs (fewer than 1000); potential doublets with excessive gene expression (more than 10,000) and high UMIs (more than 10,000); and possible signs of cell death or stress with elevated mitochondrial gene expression (over 10% of the total transcripts).

Normalization and scaling across samples were performed using Seurat’s ‘SCTransform’ function to adjust for batch variations (parameters: vars.to.regress = c("nCount_RNA", "percent.mito"), variable.features.n = 3000). Following this, we combined all samples and repeated the scaling and normalization process. The unified Seurat object was then subjected to clustering using the original Louvain method (25) and the top 30 PCA dimensions through Seurat's ‘FindNeighbors’ and ‘FindClusters’ functions (parameters: resolution = 0.5). The integrated and normalized matrix served as the foundation for subsequent analyses.

#### snRNA-Seq Cell Type Annotation

We identified cell types for each cluster through a meticulous manual assessment of marker gene expression. The selection of these marker genes was based on the CellMarker2.0 database and findings from prior single-cell PitNET studies.

#### snRNA-Seq Data Analysis

Genes that were differentially expressed across various cell types were detected using the FindMarkers function, which contrasts the gene expression profiles of cells within one cluster against those in other clusters. The Wilcoxon rank-sum test served as the statistical method for this comparison. A threshold of log2 fold change (FC) greater than 0.3 and a false discovery rate (FDR) below 0.05 were applied to determine the significance of differentially expressed genes (DEGs).

#### Analysis of Cell–Cell Interactions

We employed CellPhoneDB (version 2.0.1; RRID:SCR_017054) (21, 26) to explore the interactions between different cell types across various regions, focusing on identifying key ligand-receptor interactions. The interaction score is calculated as the average of the mean expression levels of ligand-receptor pairs involved in the interactions between distinct cell types.

#### Immunohistochemistry

Immunohistochemistry was performed on collected tissue specimens. Formalin-fixed, paraffin-embedded sections were stained using anti-Ki67 (Abcam AB16667)/anti-COL1A1 (Abcam AB138492)/anti-COL3A1 (Abcam AB6310) antibody. Antigen retrieval and secondary antibody incubation were carried out, followed by DAB visualization and hematoxylin counterstaining. For multiplex immunofluorescence staining, the following antibodies were employed: anti-MAPK1 (Protein tech 51068-1-AP), anti-IGF1R (Abcam AB263907), and anti-Ki67 (Abcam AB16667).

#### Histological Examination With HE Staining

Specimens from the cavernous sinus and saddle regions were fixed and processed for paraffin embedding. Sections were cut from the tissue blocks and stained with hematoxylin and eosin (HE) to visualize cellular details. The HE-stained sections were examined under a light microscope to assess cellular morphology, tissue architecture, and pathological features. This staining aided in the characterization and classification of the PitNETs.

#### Functional Experiment

Primers were listed as follows:

FOXO1 (NM_002015.4) overexpression:

Forward primer (5′ to 3′): ATGGCCGAGGCGCCTCAGGTGGT

Reverse primer (5′ to 3′): TCAGCCTGACACCCAGCTATGTG

MAPK1 (NM_002745.5) overexpression:

Forward primer (5′ to 3′): ATGGCGGCGGCGGCGGCGGCG

Reverse primer (5′ to 3′): TTAAGATCTGTATCCTGGCTGG

IGF1R (NM_000875.5) overexpression:

Forward primer (5′ to 3′): ATGAAGTCTGGCTCCGGAGGA

Reverse primer (5′ to 3′): TCAGCAGGTCGAAGACTGGGG

siRNA (MAPK1)

Sense (5′ to 3′): CACCAACCAUCGAGCAAAUTT

Antisense (5′ to 3′): AUUUGCUCGAUGGUUGGUGTT

siRNA (IGF-1R)

Sense (5′ to 3′): GCTGATGTGTACGTTCCTGAT

Antisense (5′ to 3′): ATCAGGAACGTACACATCAGC

Non-targeting control siRNA

Sense (5′ to 3′): UUCUCCGAACGUGUCACGU

Antisense (5′ to 3′): ACGUGACACGUUCGGAGAA

RT-PCR

MAPK1

Forward primer (5′ to 3′): ATTACGACCCGAGTGACGAG

Reverse primer (5′ to 3′): CCTGGCTGGAATCTAGCAGT

GAPDH

Forward primer (5′ to 3′): GGTCGGAGTCAACGGATTTG

Reverse primer (5′ to 3′): GGAAGATGGTGATGGGATTTC

IGF1R

Forward primer (5′ to 3′): TTAAAATGGCCAGAACCTGAG

Reverse primer (5′ to 3′): ATTATAACCAAGCCTCCCAC

FOXO1

Forward primer (5′ to 3′): GCTGCATCCATGGTGTTGTTGT

Reverse primer (5′ to 3′): CGAGGGCGAAATGTACTCCAGTT

### Plasmids

Full-length sequences of human MAPK1 and human IGF1R open-reading frames were obtained by performing PCR. The MAPK1 and IGF1R PCR fragment was inserted into pCDNA3.1-FLAG by the recombinant method, and their insertion was confirmed by sequence identification.

### Cell Lines

Pituitary tumor cell GH3 (ATCC CCL-82.1) was obtained from the American Type Culture Collection (ATCC). Brain fibroblast cell CP-R109 was obtained from Procell (Cat NO.: CP-R109). The cell line was routinely tested for *mycoplasma* contamination and authenticated by Short Tandem Repeat (STR) profiling.

Cells were maintained in Dulbecco’s modified Eagle’s medium (DMEM, ATCC) supplemented with 10% fetal bovine serum (FBS, Sigma-Aldrich) and 1% penicillin–streptomycin antibiotic (Sigma-Aldrich) and incubated at 37 °C and 5% CO_2_ in a humidified atmosphere in an incubator.

### Cell Culture

Cells were cultured according to the recommended media and culture system specified in the manufacturer's instructions. The cells were maintained in a sterile environment, in appropriate growth media supplemented with necessary nutrients and antibiotics. The cultures were incubated at 37 °C in a humidified atmosphere containing 5% CO_2_. Regular monitoring and passaging were conducted to maintain cell health and prevent overgrowth, ensuring optimal conditions for cell growth and experimentation.

### Cell Transfection

Plasmid transfections were performed using PEI. In the PEI transfection method, 500 μl of DMEM (serum-free medium) and the plasmid were placed in an empty EP tube, and PEI (three times the concentration of the plasmid) was added to the medium, followed by vigorous shaking. The mixture was incubated for 15 min. Meanwhile, the cell culture medium was replaced with 2 ml of fresh 10% FBS medium. After 15 min, the mixture was added to the cells, and the medium was replaced after 12 h. After 36 h, the transfection was completed, and the cells were consequently treated.

### RNA Interference

Genescript Company synthesized the siRNAs. All siRNA transfections were performed with Lipo3000 Transfection Reagent (Thermo Fisher Scientific) at 50 nM final concentration according to the manufacturer’s protocol. The siRNA-transfected GH3 cells were harvested for qPCR assays 48 h after transfection.

### Real-Time Quantitative PCR

Total RNA was extracted from cells using TRIzol Reagent (Invitrogen) according to the manufacturer’s instructions. Total RNA was reversely transcribed into first-strand cDNA using the PrimeScript RT reagent Kit with gDNA Eraser (Takara). The cDNAs were then used for real-time PCR (qPCR) on a CFX Opus 96 Real-Time quantitative PCR System (Bio-Rad) using SsoAdvanced Universal SYBR Green Supermix (Bio-Rad). GAPDH served as an internal control. The relative quantification of gene expression was analyzed by the 2-△△Ct method.

### Cell Proliferation Assay

For the cell proliferation assay, GH3 cells (1–3 × 10^3^ cells) were seeded in 96-well plates. CCK-8 solution (Beyotime Biotechnology, C0039) at the final concentration of 10% was added to the wells, and absorbance at 450 nm was measured 2 h after incubation to represent the relative cell numbers.

### IGF-1 and IGF-1R Inhibitor Treatment

For IGF-1 treatment, GH3 cells were treated with 100 ng/ml IGF-1 (MCE HY-P7018). For IGF-1R inhibitor treatment, GH3 cells were treated with 10 μM IGF-1R inhibitor (MCE HY-P169600).

### Co-culture System

We performed indirect coculture of CAFs and GH3 cell lines using a 6-well transwell plate under normoxia. For the investigation of cell proliferations, four groups of GH3 cells (G1: GH3 cells transfected with an empty vector, G2: GH3 cells transfected with an IGF-1R overexpressed vector, G3: IGF-1R overexpressed GH3 cells treated with an IGF-1R inhibitor, G4: GH3 cells transfected with IGF-1R siRNA) were seeded in the lower chamber, whereas CAFs were in the upper chamber. The ratio of GH3 cells to CAFs was 1:1. After incubation for 24 h, GH3 cells were harvested for the detection of cell proliferation-related proteins and the CCK-8 assay.

### Polymerase Chain Reaction Assay

PCR analysis was conducted to assess the gene expressions of IGF1, IGFBP1, and IGF1R. RNA was extracted from the collected tissue specimens using standardized protocols. Complementary DNA (cDNA) was synthesized from the extracted RNA through reverse transcription. Specific primers for IGF1, IGFBP1, and IGF1R were designed and used in separate PCR reactions along with the cDNA template, PCR master mix, and appropriate thermal cycling conditions. After amplification, the PCR products were analyzed to determine the relative quantity of the target genes.

### CCK-8 Assay

Cell viability was assessed using CCK-8 assay after overexpressing IGF1R and/or IGF1. Cells were transfected with respective plasmids and incubated for specified periods. CCK-8 solution was added, and absorbance was measured at 450 nm using a microplate reader. This enabled the quantification of cellular proliferation following IGF1R and/or IGFBP1 overexpression.

### Cellular Proteome

#### Sample Preparation

Following transfection, IGF1R-OE, FOXO1-OE, and other relevant cells were harvested. The cell pellets were washed sequentially with PBS and centrifuged at 15,000×*g* for 10 min per wash until the supernatant was clear to remove residual culture medium.

Cell lysis was performed using 8 M urea lysis buffer (8 M urea, 100 mM Tris-HCl, pH 8.0) supplemented with protease and phosphatase inhibitors (Thermo Scientific), followed by sonication for 1 min (15% amplitude, 1 s on/1 s off). The lysates were then centrifuged at 15,000×*g* for 10 min to remove debris, and the protein concentration of the supernatant was determined by Bradford assay.

For digestion, proteins were first denatured at 37 °C for 30 min, reduced with 10 mM DTT at 37 °C for 30 min, and alkylated with 20 mM iodoacetamide (IAA) in the dark at room temperature for 20 min. Excess IAA was quenched with 10 mM DTT for 5 min at room temperature.

Protein digestion was carried out using the filter-aided sample preparation (FASP) method. Briefly, samples were loaded onto a 30-kDa Microcon filter (Millipore) and centrifuged at 14,000×*g* for 20 min. The retained material was washed twice with 300 μl of 8 M urea in 100 mM Tris (pH 8.0) and subsequently equilibrated with 200 μl of 100 mM NH_4_HCO_3_. Trypsin was added at a 50:1 protein-to-enzyme ratio (w/w), and digestion proceeded at 37 °C for 16 h. The resulting peptides were collected by centrifugation, dried in a vacuum concentrator (Thermo Scientific), and 10% of the peptides were subjected to subsequent proteomic analysis. Mass spectrometric analysis of the tryptic peptides and subsequent protein database search were performed as previously described for the tissue proteomic analysis. For each cell line, three biological repeats were performed.

#### Differential Proteomic Analysis

For the proteomic data obtained from different cell groups, differentially expressed proteins were screened using the criteria of fold change (FC) > 1.5 and Wilcoxon *p* < 0.05. The expression patterns of representative molecules were visualized using GraphPad.

#### Transwell Assays

To assess tumor cell migration and invasiveness, we employed Transwell assays as a robust experimental approach. Briefly, CAFs derived from cavernous sinus tumors were co-cultured with GH3 cells in the upper chamber of the Transwell system, separated by a porous membrane. After a designated incubation period, migrated cells on the lower surface of the membrane were fixed, stained, and counted, offering a quantitative evaluation of their migratory and invasive capabilities.

#### Subcutaneous Xenograft Tumor Formation

Female BALB/c nude mice (4–6 weeks old) were subcutaneously injected in the right flank with 1 × 10^6^ GH3 rat pituitary tumor cells (purchased from the Cell Resource Center, Institute of Basic Medical Sciences) suspended in 100 μl of serum-free medium. All animal procedures were approved by the Ethics Committee of Zhongshan Hospital, Fudan University. Tumors were harvested 2 weeks post-injection for subsequent experiments.

#### Statistical Analysis

To ensure the rigor and reproducibility of our findings, we employed a range of statistical methods. Specifically, data were expressed as mean ± standard deviation (SD) unless otherwise stated. Comparisons between groups were made using Student's *t* test or one-way analysis of variance (ANOVA) followed by Tukey's post-hoc test, as appropriate. Correlation analyses were performed using Pearson's correlation coefficient. *p*-values less than 0.05 were considered statistically significant, and all tests were two-tailed. Multiple comparisons were adjusted using appropriate statistical methods to control for type I errors. All statistical analyses were performed using GraphPad Prism software, and the specific tests used are detailed in the figure legends and accompanying tables.

## Results

### Patient Cohort and Clinical Characteristics

**Fig. 1:**
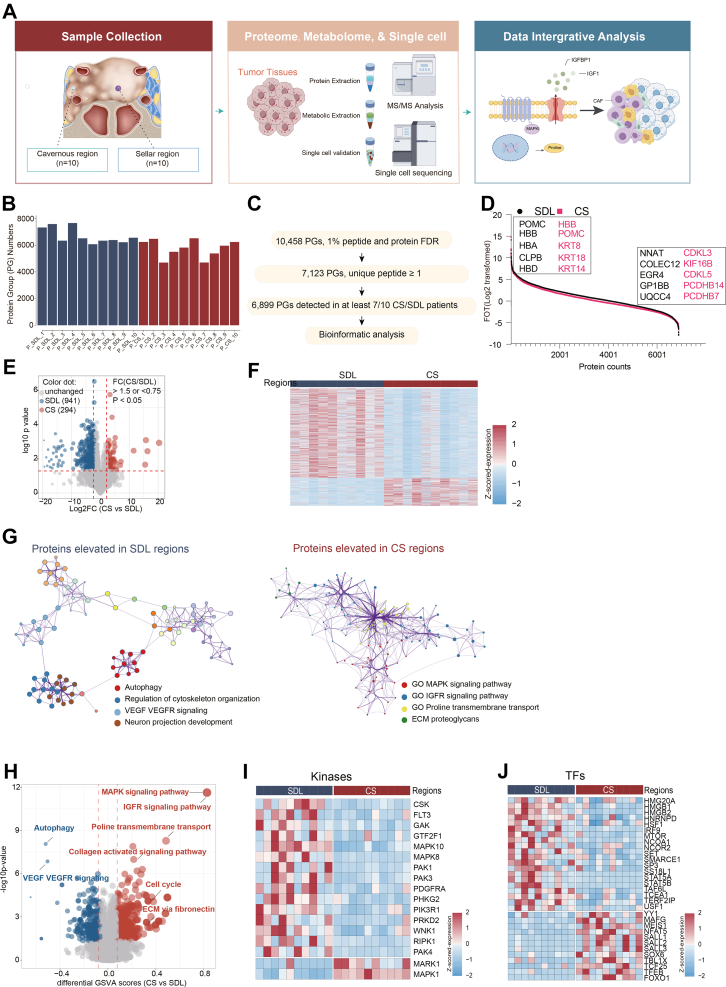
**Overview of the proteomic landscape of PitNETs.***A*, the experimental workflow. *B*, the barplots showcasing the detected protein numbers of each sample. *C*, proteomic datasets were filtered at different levels for various statistical analyses. *D*, dynamic range of the proteome of CS and SDL samples, based on protein abundance (Log 2(FOT)). The different colors represent proteins in CS and SDL. *E*, the volcano plot of the *p*-values vs the log2 protein abundance difference in CS compared to SDL samples. Significantly upregulated and downregulated proteins are highlighted in *red* and *blue*. *F*, subgroups are identified based on the proteomic data of all samples by K-means consensus clustering upon their abundance. k = 2 was selected, and consensus clustering was based on 1000 resampled datasets. *G*, the pathways enriched by proteins were significantly elevated in CSs (*up*) and SDLs (*down*). *H*, the volcano plot of the *p*-values vs the differential GSVA scores in CS compared to SDL samples. Significantly upregulated and downregulated GSVA scores are highlighted in *red* and *blue*. *I*, the heatmap presenting kinases significantly elevated in CSs and SDLs. *J*, the heatmap presents TFs significantly elevated in CSs and SDLs.

We collected a total of 20 paired specimens from the saddle (SDL) and cavernous sinus part (CS) of PitNETs of 10 patients, and three normal pituitaries (NPs) from adult donors were collected. The specimens were subjected to proteomic and metabolomic profiling to investigate the underlying molecular mechanisms. Integrated analysis of multi-omics data revealed potential pathways involved, which were further validated using single-cell transcriptomic data (Fig. 1*A*). A total of 10 individuals, consisting of six female participants with an average age of 40.10 ± 9.69 years, were included in the study. These PitNETs were all classified as Knosp grade 4. The endocrine profiles and immunohistochemical findings are presented in the accompanying table (Supplemental Table S1).

### Proteomic Data Revealed Differences Between Saddle and Cavernous Sinus Tumours

To systematically characterize molecular differences between pituitary tumors arising in the saddle (SDL) and cavernous sinus (CS), we performed mass spectrometry–based proteomic profiling on 23 specimens—10 SDL tumors, 10 matched CS tumors, and three normal adult pituitary tissues (NPs) (Fig. 1*A*). A total of 10,458 proteins were identified, and after stringent quality control (Methods, Fig. 1, *B* and *C*), 6899 high-confidence proteins were retained for analysis. The dynamic range of protein abundance was comparable between groups (Fig. 1*D*), supporting data reliability. Differential expression analysis revealed 1248 significantly altered proteins between CS and SDL tumors (Fig. 1*E*). Unsupervised consensus clustering (K-means, *k* = 2) robustly segregated all tumor samples into two distinct subgroups (Supplemental Fig. S1), a finding validated across 1000 resampling iterations. Functional interpretation showed that CS-enriched proteins were associated with MAPK and IGF1R signaling, proline transmembrane transport, and ECM proteoglycan metabolism, whereas SDL-enriched proteins were linked to autophagy, cytoskeleton organization, VEGFR signaling, and neuron projection development (Fig. 1, *F* and *G*), indicating anatomically distinct biological programs. At the pathway level, Gene Set Variation Analysis (GSVA) confirmed coordinated shifts in activity, with multiple pathways significantly up- or downregulated (*p* < 0.05; Fig. 1*H*). Finally, kinase activity profiling and transcription factor enrichment analyses revealed non-overlapping regulatory networks: CS tumors exhibited activation of growth factor–related regulators, while SDL tumors showed enrichment for factors involved in structural remodeling and neural development (Fig. 1, *I* and *J*). Together, these results demonstrate that SDL and CS pituitary tumors possess fundamentally different proteomic landscapes, driven by location-specific signaling, functional pathways, and regulatory mechanisms.

### The IGF1–IGF1R–MAPK Axis Drives Tumor Cell Proliferation in Cavernous Sinus Tumor

**Fig. 2:**
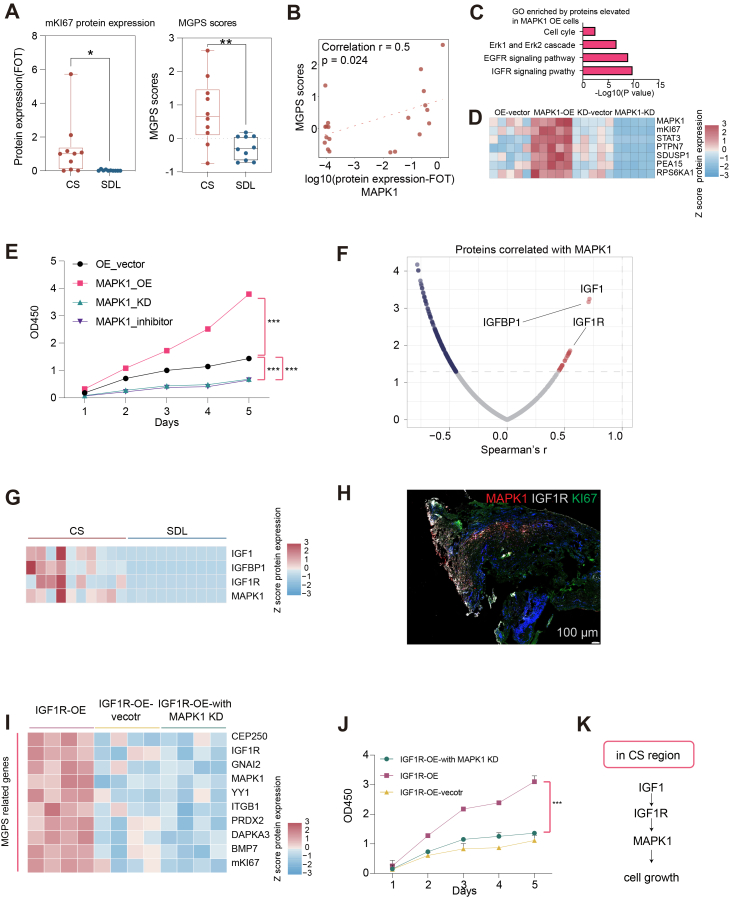
**The IGF1-IGF1R-MAPK axis promotes tumor cell growth.***A*, the boxplots showing the comparison of protein expression of MKI67 and Cell cycle features (MGPS scores) between CS and SDL. *B*, the scatter plot indicated the Spearman’s correlation between MAPK1 protein expression and MGPS scores. *C*, Bar plot showing the GO enriched by proteins elevated in MAPK1 OE cells. *D*, heatmap showing the represented proteins that altered across cells under different treatments. *E*, proliferation of cells associated with various treatments (n = 5, mean ± SEM, two-sided Student *t* test). *F*, volcano plot indicating ligands and receptors that are associated with protein expression of MAPK1. *G*, heatmap indicating represented ligands and receptors that were elevated in CS samples. *H*, the IF staining indicated enrichment of MAPK1 (*Red*), KI67 (*Green*), and IGF1R (*White*) in CS samples. Scale bar 100 μm. (*I*) Heatmap showing the represented proteins that altered across cells under different treatments. *J*, proliferation of cells associated with various treatments (n = 5, mean ± SEM, two-sided Student *t* test). *K*, the systematic diagram summarizes the impact of the IGF1R-MAPK1 axis in promoting tumor cell proliferation.

Given that cavernous sinus (CS) tumors exhibited higher MKI67 expression and elevated cell cycle activity (as reflected by MGPS scores) compared to saddle (SDL) tumors (Fig. 2*A*), we hypothesized that MAPK signaling contributes to this proliferative phenotype. Consistent with this, MAPK1 protein levels positively correlated with MGPS scores across samples (Fig. 2*B*), suggesting a functional link between MAPK1 and cell cycle progression. To test this, we modulated MAPK1 expression in GH3 pituitary tumor cells: we generated MAPK1-overexpressing (MAPK1-OE) and MAPK1-knockdown (MAPK1-KD) lines using an overexpression vector or siRNA, respectively, with empty vector and scramble siRNA as controls (Supplemental Fig. S2*A*, Methods). RT-qPCR and proteomic validation confirmed successful modulation of MAPK1 (Supplemental Fig. S2*B*). Strikingly, overexpression of MAPK1 upregulated key proliferation markers—including MKI67 and STAT3—at the protein level, whereas MAPK1 knockdown reduced their expression (Fig. 2, *C* and *D*). Accordingly, MAPK1-OE cells displayed significantly higher MGPS scores than OE-vector controls, while MAPK1-KD cells showed lower scores than KD-vector controls (Supplemental Fig. S1*C*). These molecular changes translated into functional differences: CCK-8 assays demonstrated that MAPK1-OE enhanced cell proliferation, whereas MAPK1-KD suppressed it (Fig. 2*E*).

Notably, ligand–receptor interaction analysis identified strong associations between MAPK1 expression and CS-enriched ligands/receptors (Fig. 2, *F* and *G*). Immunofluorescence staining confirmed co-enrichment of MAPK1, MKI67, and IGF1R specifically in CS tumor tissues (Fig. 2*H*). Targeted inhibition of this axis—via knocking down expression of MAPK1 in IGF1R overexpression cells—significantly reduced cell proliferation rates (Fig. 2, *I* and *J*). Collectively, these findings support a model in which the IGF1–IGF1R–MAPK1 signaling axis promotes tumor cell proliferation in CS through coordinated activation of cell cycle regulators, phospho-dependent signaling cascades, and autocrine/paracrine ligand–receptor networks (Fig. 2*K*).

### Metabolomics Unveils the Close Link Between Proline Metabolism and CAF Activity in Cavernous Sinus Tumors

**Fig. 3:**
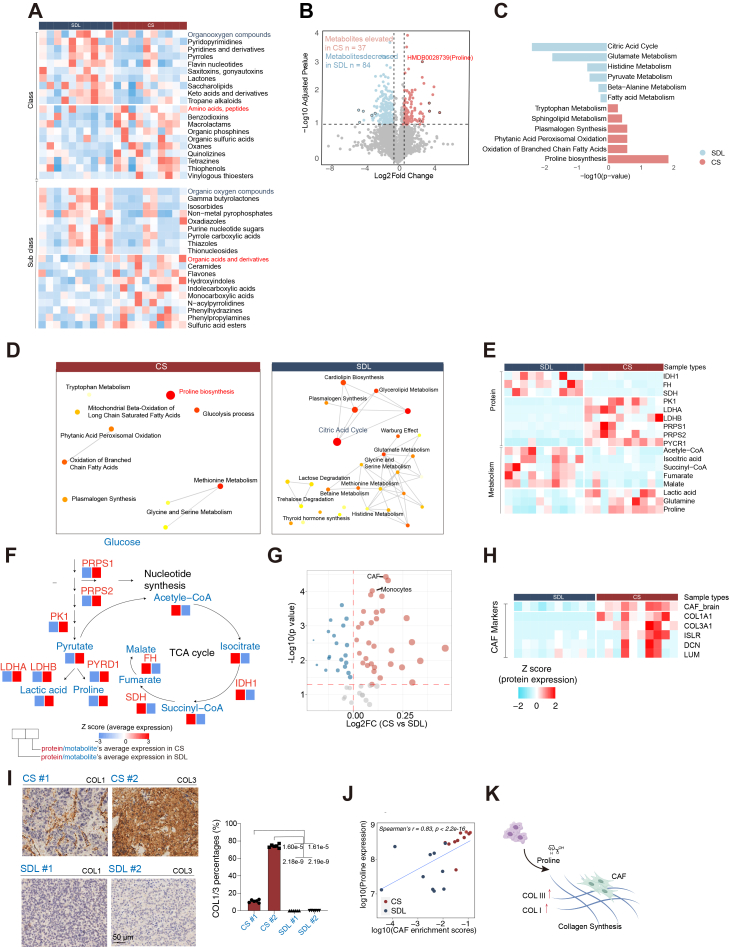
**Metabolome landscape of pituitary neuroendocrine tumors located in CS and in SDL.***A*, the heatmap presenting the abundance of metabolites detected across the samples. Metabolites are sorted by classes and subclasses to which they belong. *B*, the volcano plot indicating the metabolites elevated in CS and SDL, accordingly. *C*, the bar plots showcase metabolic pathways that were elevated in SDL and CS. *D*, the networks presenting metabolic pathways that were elevated in SDL and CS. Colors present the pathway enrichment *p*-value (low to high from *yellow* to *red*), and the circle sizes indicate the number of metabolites in each pathway. *E*, the heatmap presents the abundance of metabolites and proteins detected across the samples. *F*, the pathway networks presenting the representative proteins and metabolites in the TCA cycle and glycolysis. The heatmap below indicates the comparison of proteins and metabolites between SDLs and CSs. *G*, the volcano plot of the *p*-values vs the differential cell type enrichment scores in CS compared to SDL samples. Significantly upregulated and downregulated cell type enrichment scores are highlighted in *red* and *blue*. (*H*) The heatmap presents the abundance of fibroblast-specific proteins detected across the samples. *I*, immunohistochemistry of COL1, COL3 in CS and SDL samples (analyzed patients: n = 4). Scale bar = 100 μm. The barplots showcase the quantification of COL1, COL3-positive cells in CS and SDL samples. *J*, the scatter plot indicating the association between Fibroblast enrichment scores and abundance of Proline. Colors present two types of samples (CS: *red*, SDL: *navy*). *K*, schematic model illustrating proline-driven promotion of CAF activity, leading to increased collagen production and fibrosis in the tumor microenvironment.

Given the observed upregulation of IGF1 in cavernous sinus (CS) tumors, we further explored whether the distinct tumor microenvironments between CS and saddle region (SDL) tumors contribute to this IGF1 elevation. To address this, we first performed untargeted metabolomic profiling to compare metabolite profiles between CS and SDL samples. The abundance of all detected metabolites across all samples was visualized using a hierarchical clustering heatmap (Fig. 3*A*), where metabolites were grouped by their chemical classes and subclasses to clearly display distribution patterns and inter-sample differences. Differential metabolite expression between CS and SDL samples was analyzed using volcano plots (Fig. 3*B*), with statistical significance defined as *p* < 0.05; this analysis revealed significant elevations in specific metabolites in CS samples, with proline showing one of the most prominent increases.

To identify metabolic pathways dysregulated between the two regions, pathway enrichment analysis was performed, and the results were summarized in bar plots (Fig. 3*C*) and network diagrams (Fig. 3*D*). In these visualizations, pathway enrichment *p*-values were represented by a color gradient, and the number of metabolites associated with each pathway was reflected by node size, clearly highlighting pathways preferentially enriched in CS or SDL samples. A combined heatmap (Fig. 3*E*) integrated metabolite and protein abundance data, enabling the identification of potential correlations between these two molecular layers and providing clues for cross-omics interactions.

Representative metabolic pathways, including the tricarboxylic acid (TCA) cycle and glycolysis (both closely related to tumor metabolic reprogramming), were further detailed in pathway networks (Fig. 3*F*); the heatmap below these networks showed the differential expression of key proteins and metabolites involved in these pathways between CS and SDL samples, clarifying the molecular basis of metabolic differences. Cell type-specific enrichment scores, calculated to link metabolite changes to specific cell populations, were compared using a volcano plot (Fig. 3*G*), which identified significantly upregulated and downregulated cell types in CS versus SDL samples, with fibroblasts showing a marked upregulation in CS.

To further characterize fibroblast-related molecular changes, a heatmap (Fig. 3*H*) was generated to display the abundance patterns of fibroblast-specific proteins across all samples, confirming elevated fibroblast activity in CS tumors. Immunohistochemical (IHC) staining was performed to validate the expression of collagen proteins (COL1 and COL3, key markers of CAF-mediated fibrosis) in CS and SDL samples (n = 4 per group), with quantification of positive cells presented in bar plots (Fig. 3*I*); this confirmed significantly higher COL1 and COL3 expression in CS samples, consistent with enhanced CAF activity. Finally, a scatter plot (Fig. 3*J*) demonstrated a positive association between fibroblast enrichment scores and proline abundance, with CS samples (red) and SDL samples (navy) clearly distinguished by color. Collectively, these metabolomic and validation data illustrate a clear relationship: higher proline levels in the CS tumor microenvironment stimulate cancer-associated fibroblasts (CAFs) to produce more collagen fibers, thereby creating a supportive niche that promotes tumor cell proliferation and invasion (Fig. 3*K*).

### Single-Cell Analysis Unveils the Crucial Role of CAFs and IGF1-IGF1R Interaction in CS Tumors

**Fig. 4:**
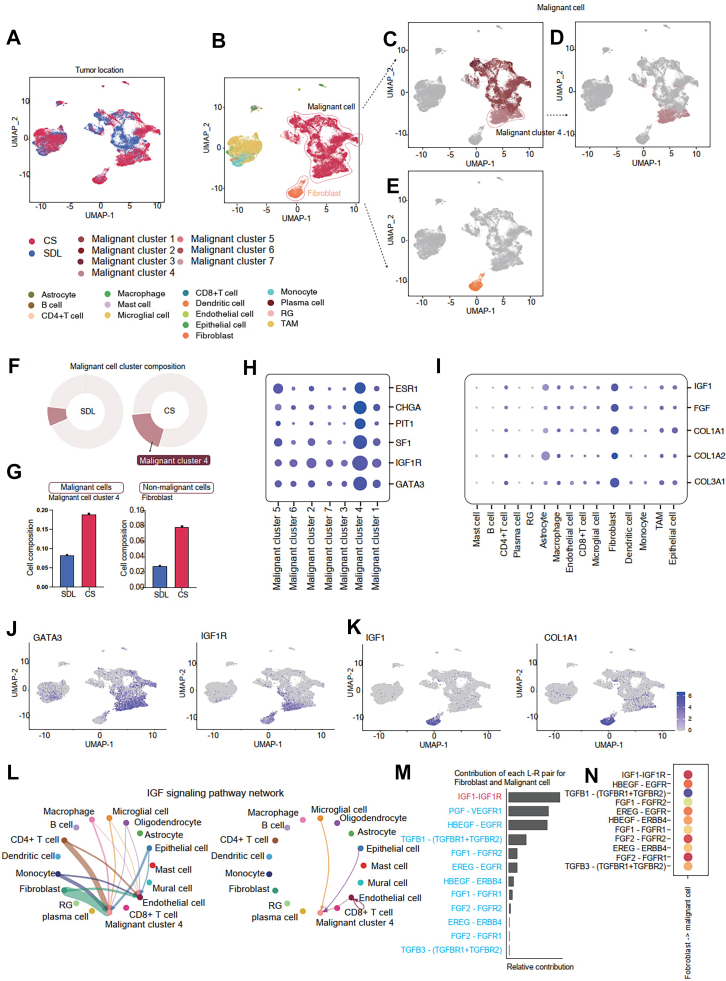
**Single-cell landscape of pituitary neuroendocrine tumors located in CS and in SDL.***A*, uniform manifold approximation and projection (UMAP) plot showing a total of 33,084 cells from CS samples (*red*, 15,176 cells) and SDL samples (navy, 17,908 cells). *B*, UMAP plot presenting different types of malignant (18,308) and non-malignant cells (14,776) across all CS and SDL samples. *C*, UMAP plot presenting 7 clusters of malignant cells, including cluster 1 (6429), cluster 2 (2345), cluster 3 (3575), cluster 4 (2121), cluster 5 (1246), cluster 6 (561), and cluster 7 (2031) across all CS and SDL samples. *D*, UMAP plot presenting cluster 4 of malignant cells (2121) across all CS and SDL samples. *E*, UMAP plot presenting Fibroblast (2806) across all CS and SDL samples. *F*, Frequencies of cellular states for CS sample and SDL sample groups from (*D*). *G*, Bar plots indicated the comparison of cluster 4 frequencies between SDL and CS. *H*, Bubble plot presenting the top-featured gene expression of cluster 4 in malignant clusters. *I*, Bubble plot presenting the top featured gene expression of Fibroblast across all cell types. (*J*) UMAP plots showing the expression of GATA3, IGF1R across all cell types. (*K*) UMAP plots showing the expression of IGF1 and COL1A1 across all cell types. *L*, Circle plot visualizing the inferred IGF signaling pathways between CSs and SDLs. Circle sizes are proportional to the number of cells in each cell group, and the thickness of the flow represents the interaction weight. *M*, Bar plot indicated the relative contributions of ligand-receptor pairs between fibroblasts and malignant cells. *N*, Bubble heatmap showcasing the principal mediating proteins and interactions that facilitate communication between malignant cell cluster 4 and fibroblast.

To further investigate the distinct tumor microenvironments underlying the heightened proliferative features of CS tumors, we performed single-cell transcriptomic analysis on samples from both CS and SDL regions. We also concurrently incorporated external, well-annotated single-cell RNA sequencing data of CS tumors with clear cavernous sinus invasion, based on which we conducted integrated analyses. After initial quality control to remove low-quality cells, doublets, and debris, the final dataset comprised 33,084 cells, including 15,176 from CS samples and 17,908 from SDL samples, as visualized in the uniform manifold approximation and projection (UMAP) plot (Fig. 4*A*).

Using unsupervised clustering, malignant cells (18,308 cells) and major microenvironmental cell populations (14,776 cells) were distinguished based on characteristic marker genes (Methods). We identified 15 non-malignant cell clusters: astrocyte (GFAP, S100B, ALDH1L1), B cell (CD79A, CD79B, CD20), CD4+ T cell (CD3D, CD4), macrophage (CD68, CD163, CCL13), mast cell (TPSAB1, FCER1A, MS4A2), microglia (TMEM119, SLC2A5, TGFBR1, CSF1R), CD8+ T cell (CD8A, GZMA, GZMB), dendritic cell (HLA-DRA, HLA-DRB1, HLA-DQB1), endothelial cell (PECAM1, CDH5, VWF), epithelial cell (KRT8, TTR, FOXJ1), fibroblast (COL1A1, COL1A2, COL1A3, FGF), monocyte (CD14, S100A8, VCAN), plasma cell (IRF4, PRDM1, XBP1), TAM (AIF1, CD68, CSF1R), and radial glial cells (SOX2, NES) (Fig. 4, *B* and *E*).

For malignant cells, seven distinct subclusters were identified: cluster 1 (6429 cells), cluster 2 (2345 cells), cluster 3 (3575 cells), cluster 4 (2121 cells), cluster 5 (1246 cells), cluster 6 (561 cells), and cluster 7 (2031 cells) (Fig. 4, *C* and *D*). Comparative analysis revealed differences in malignant and non-malignant cell distributions between CS and SDL samples. Notably, CS samples contained a higher proportion of malignant cells from cluster 4 (CS: 18.90% *versus* SDL: 8.27%) and fibroblasts (CS: 18.07% vs. SDL: 7.8%) (Fig. 4, *F* and *G*).

Differential expression analysis of malignant cluster four showed enrichment for genes involved in the IGF1-IGF1R signaling axis (ESR1, CHGA, PIT1, SF1, IGF1R, and GATA3) (Fig. 4*H*). In addition to known fibroblast markers (COL1A1, COL1A2, COL1A3, FGF), elevated expression of IGF1, the ligand for IGF1R, was observed in fibroblasts (Fig. 4, *I*–*K*).

Further cell communication analysis indicated that the most prominent ligand-receptor pair in CS samples was IGF1-IGF1R (Fig. 4, *L* and *M*). Moreover, communication mediated by the IGF signaling pathway between fibroblasts and malignant cluster four cells was significantly stronger in CS samples compared to SDL samples (Fig. 4*N*).

Collectively, these single-cell analyses identified fibroblasts as a major component of the CS tumor microenvironment and uncovered IGF1-IGF1R-mediated crosstalk between fibroblasts and a specific subpopulation of malignant cells (cluster 4) in CS tumors, providing a mechanistic basis for the enhanced proliferation and invasiveness observed in this region.

#### IGF1 Upregulation by FOXO1 in CAFs Promotes Tumor Cell Proliferation

**Fig. 5:**
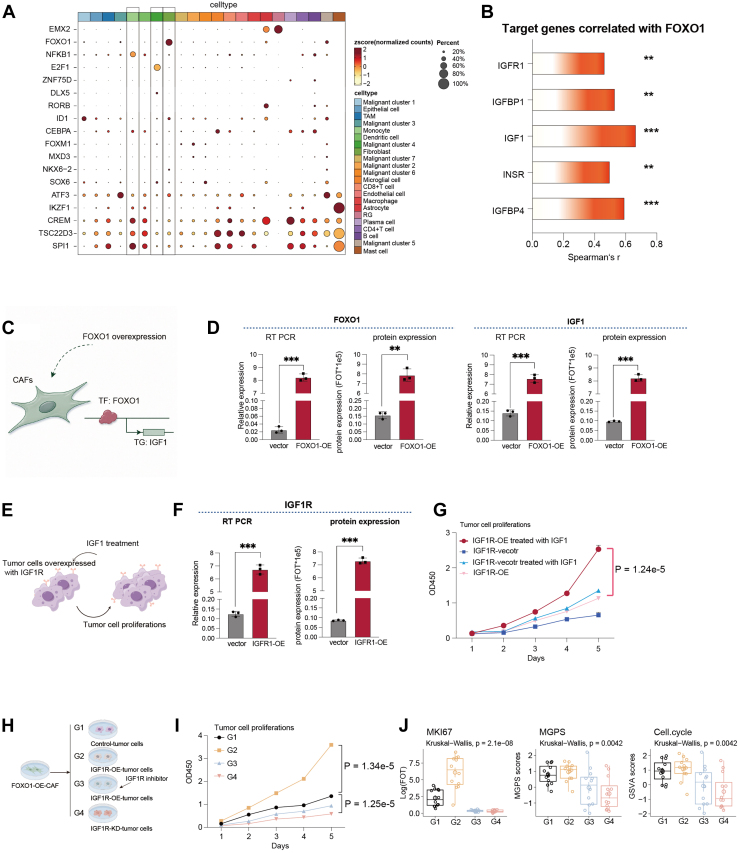
**Transcription factor analysis and validation.***A*, Transcription factor analysis identifies key transcription factors specific to CAFs, Tumor-IGF1R, and Monocytes. *B*, correlation analysis between the CAF transcription factor FOXO1 and its target genes. *C*, schematic diagram illustrating how FOXO1 overexpression can promote CAF secretion of IGF1. *D*, *Left*: Validation of FOXO1 overexpression at both the RNA and protein levels; *Right*: FOXO1 overexpression leads to increased IGF1 levels at both the RNA and protein scales. *E*, schematic model showing that IGF1 overexpression can promote cell proliferation in cells with IGF1R overexpression. *F*, validation of IGF1R overexpression at both the RNA and protein levels. *G*, CCK-8 assay detecting cell proliferation demonstrates that tumor cells with overexpression of IGF1R treated with IGF1 exhibit the fastest proliferation rate. *H*, schematic diagram summarizing the proposed co-cultivation systems utilized to confirm the interaction between FOXO1-OE-CAFs and tumor cells. *I*, CCK-8 assay detecting cell proliferation demonstrates that tumor cells under different treatments. *J*, the comparison of MKI67 expression, MGPSs, and the cell cycle enrichments among different groups.

To investigate the reason behind the elevated secretion of IGF1 by CAFs, we performed a transcription factor analysis within our single-cell transcriptomic data (Methods). The results revealed elevated AUC values of FOXO1 in CAFs, E2F1 in IGF1R-positive tumor cells, and NFKB1 in Monocytes (Fig. 5*A*). Correlation analysis demonstrated a strong association between the transcription factor FOXO1 and its target genes, IGF1 (Fig. 5*B*). These findings suggest that the expression of FOXO1 promotes the transcription of IGF1 (Fig. 5*C*). To validate this, we overexpressed FOXO1 in CAFs (Methods) and observed an increase in IGF1 expression (Fig. 5*D*). Furthermore, we generated stable cell lines with overexpressed IGF1R and subsequently treated with IGF1 (Fig. 5, *E* and *F*). We then evaluated the cell proliferation rates and found that IGF1R-overexpressed tumor cells that were treated with IGF1 showed the most significantly elevated proliferation (Fig. 5*G*).

To further demonstrate our assumptions, we constructed a co-cultivation system that cultivated tumor cells (G1: control pituitary tumor cells; G2: IGF1R-overexpressed pituitary tumor cells; G3: IGF1R-overexpressed pituitary tumor cells treated with IGF1R inhibitor; G4: IGF1R-KD pituitary tumor cells) with FOXO1-overexpressed CAFs (Fig. 5*H*). We then evaluated the tumor cell proliferation rate and found that IGF1R-overexpressed pituitary tumor cells co-cultivated with FOXO1-overexpressed CAFs showed the fastest tumor cell proliferation rate (Fig. 5*I*). To confirm our finding, we also applied proteomic analysis. The results indicated tumor cell proliferation markers like MKI67, the cell proliferation scores MGPS, and the GSVA scores of cell cycle, all showed elevation in G2 group (Fig. 5*J*). Taken together, these data implicate that CAFs promote tumor growth by secreting IGF1, which acts on tumor cells expressing IGF1R, thereby enhancing their proliferation.

#### Cross-Talk Between CAFs and Tumor Cells

**Fig 6:**
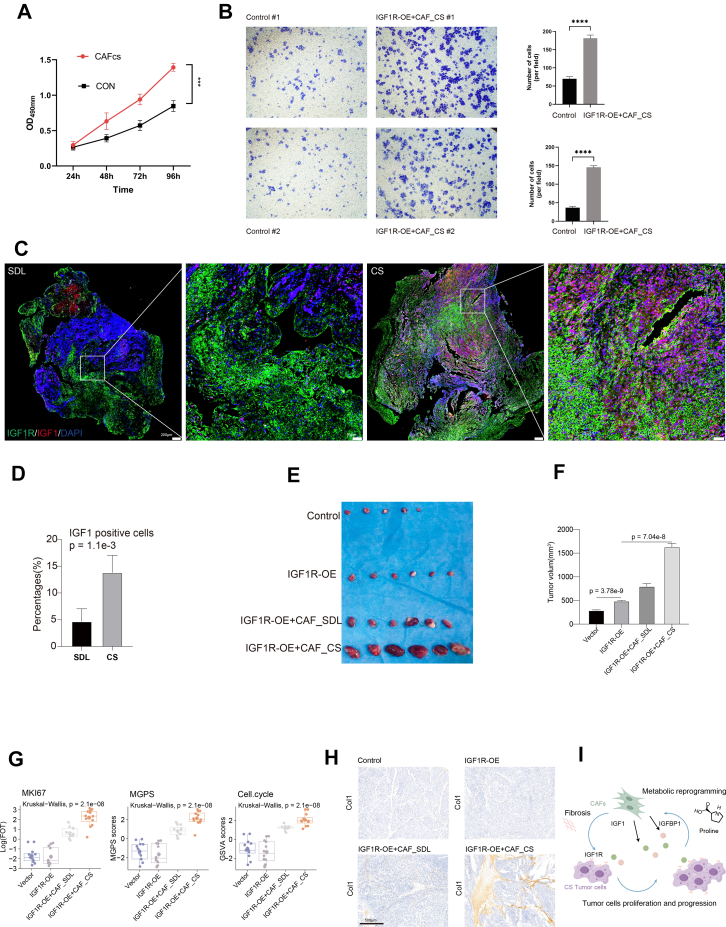
**Crosstalk between CAFs and tumor cells.***A*, Co-culture of CAFs derived from cavernous sinus tumors with GH3 cells, assessing cell proliferation using the CCK-8 assay. *B*, Transwell assays examining tumor migration and invasiveness, revealing an increase in aggressive phenotypes upon co-culture with CAFs. *C*, immunofluorescence staining of tumor samples from the CS region shows increased IGF1 (*red*) compared to SDL samples, with IGF1R in *green* and nuclei in *blue*. *D*, percentage of IGF1-positive cells in SDL and CS. *E-F*, subcutaneous tumorigenesis experiments in nude mice demonstrate that CAFs enhance the tumor volume of GH3 cells, with CAFs from CS exhibiting a stronger tumor growth-promoting ability than those from SDL. *G*, the boxplots indicated the expression of MKI67, MGPSs, and the enrichment of cell cycle among groups. *H*, the IHC staining presented the comparison of collagen expression among groups. *I*, schematic representation of the direct crosstalk between CAFs and tumor cells, highlighting the complex interplay between these cellular components in tumor progression.

To elucidate the influence of CAFs on tumor cell proliferation, we co-cultured CAFs derived from the cavernous sinus with GH3 cells and conducted Transwell assays. Our results unequivocally demonstrate that the presence of CAFs enhances the proliferation (Fig. 6*A*), migration, and invasiveness (Fig. 6*B*) of GH3 cells. Further investigation through immunofluorescence staining of tumor samples from the CS region disclosed a marked increase in IGF1 expression (depicted in red) when juxtaposed with those from the SDL region, with IGF1R visualized in green and cell nuclei in blue (Fig. 6*C*). This differential staining underscores the distinct molecular milieu in the CS region. Subsequent subcutaneous tumorigenesis experiments in nude mice corroborated the potentiating effect of CAFs on the tumor volume of GH3 cells, with CAFs isolated from the CS region showcasing a more robust capacity to foster tumor growth compared to those from the SDL region (Fig. 6, *D* and *E*).

Moreover, by constructing xenograft tumor transplantation (Methods), we found that xenograft growth of IGF1R-overexpressed tumor cells co-cultivated with CAFs from CS regions was the fastest (Fig. 6, *E* and *F*). Consistently, the tumor cell proliferation marker MKI67, the MGPS scores, and the enrichment of cell cycle, all showed elevation in xenograft tumors of IGF1R-overexpressed tumor cells co-cultivated with CAFs from CS regions (Fig. 6*G*). Also, these xenograft tumors showed enrichment of collagens (Fig. 6*H*). These results confirm that the cellular communications of CAFs-IGF1R-overexpressed tumor cells mediated by the IGF1-IGF1R signaling pathway might be responsible for the malignant tumor cell growth in CSs (Fig. 6*I*).

## Discussion

Due to the limitations in surgical instruments and techniques in the past, obtaining specimens from the cavernous sinus, especially for the fibrous tumors, has been extremely challenging (27). Consequently, there has been a scarcity of research focusing on the differences between the cavernous sinus and saddle regions in invasive PitNETs. However, studying these differences holds significant implications for managing invasive PitNETs that are difficult to treat comprehensively. To our knowledge, our team is the first to investigate the disparities between the cavernous sinus and saddle regions in invasive PitNETs in multi-omics aspects. The findings of our study reveal distinct variations between these regions in invasive PitNETs, elucidate the biological characteristics of tumors in the cavernous sinus, and identify possible pathways contributing to their invasiveness. These discoveries provide a foundation for the development of novel therapeutic targets in the future.

Existing literature broadly supports MAPK1 (ERK2) as a key proliferative driver in PitNET. Studies confirm that sustained ERK/MAPK pathway activation—triggered by growth factors, receptor tyrosine kinases (e.g., EGFR), or mutations (e.g., BRAF V600E in papillary craniopharyngioma)—promotes cell-cycle progression by upregulating cyclins (e.g., Cyclin D1) and repressing apoptosis regulators. For instance, in non-functioning pituitary adenomas (NFPAs), elevated MAPK1 expression correlates with tumor growth and invasiveness, aligning with our observation that MAPK1 enhances proliferation. Cross-talk between MAPK and other pathways (e.g., cAMP/PKA, PI3K/Akt) underscores its central role in pituitary tumor biology (28, 29, 30, 31). In our study, we linked MAPK1 overexpression to tumor location (SDL *versus* CS). Furthermore, proteomics data also pointed to the activation of the IGF1R-MAPK1 pathway as a key driver of increased invasiveness in the cavernous sinus region. For now, no studies have directly tied MAPK1 activity to anatomic zonation. Thus, our findings for the first time illustrated that MAPK1 might be the key regulator that contributed to the elevated tumor cell proliferations of PitNET located in CS.

Besides, our findings refine precision potential medicine approaches for PitNETs. While existing literature explores MAPK inhibitors (e.g., MEK/ERK blockers) or microRNAs (e.g., miR-16 targeting MEK1), therapies remain location-agnostic. Our data imply CS tumors may respond better to MAPK inhibitors, whereas SDL tumors might require combinatorial strategies (e.g., MMP/VEGF inhibitors). Further, region-specific activators of MAPK1 (e.g., intrasellar pressure) could be therapeutically modulated (e.g., via MST4/p38 regulators).

From our analysis of single-cell transcriptomic data, two intriguing findings emerge. Firstly, the cavernous sinus tumors display a remarkable fibroblast content of approximately 34%, which stands in stark contrast to the significantly lower proportion found in saddle region tumors. Secondly, within the cavernous sinus, a distinct subpopulation of tumor cells expressing IGF1R is identified, accounting for approximately one-third of the total tumor cell population. What does the marked abundance of CAFs portend? Previous research has illuminated the multitude of mechanisms by which CAFs can facilitate tumor growth and enhance invasiveness, ranging from paracrine signaling to extracellular matrix remodeling (32, 33, 34, 35). What role do CAFs play in the context of cavernous sinus tumors? Our analysis of transcription factors from single-cell transcriptomic data reveals a notable finding: CAFs exhibit high expression of FOXO1. This elevated expression of FOXO1 drives the secretion of IGFBP1 and IGF1, key growth factors implicated in tumor progression. Specifically, these data suggest that CAFs, through the action of FOXO1, can enhance the proliferation of IGF1R-positive tumor cells by secreting IGFBP1 and IGF1.

Metabolomics data reveal a striking difference in proline metabolism between the cavernous sinus region and the saddle region, with proline standing out as a key metabolite exhibiting significant disparities. Furthermore, our findings offer compelling evidence for a pronounced Warburg effect in cavernous sinus tumors, as evidenced by the marked increase in glycolytic activity. These observations highlight the critical role of metabolic reprogramming in the pathogenesis of cavernous sinus tumors and underscore the importance of regional metabolic variations in shaping the tumor microenvironment. The xCell analysis (36) from proteomics data suggested that CAFs might play a significant role in cavernous sinus tumors. We elucidated the relationship between proline and CAFs, as well as their association with fibrosis. A study (37) has demonstrated that high expression of proline promotes the secretion of collagen by CAFs, leading to increased tumor aggressiveness. In this process, the key enzyme involved in proline synthesis is PYCR1. Our proteomics data also revealed elevated levels of PYCR1, as well as increased expression of collagen synthesis-related proteins COL1A1 and COL3A1, in cavernous sinus tumors. Moreover, metabolomics analysis detected high levels of proline expression. These findings strongly suggest that CAFs in cavernous sinus tumors enhance their collagen production capacity through metabolic reprogramming, which subsequently contributes to increased invasiveness.

Crosstalk between tumor cells and CAFs is a well-documented phenomenon (38, 39, 40), and our study identifies a specific interaction in PitNETs: CAFs secrete IGFBP1 and IGF1 via FOXO1 to promote the proliferation of IGF1R-positive tumor cells, while tumor cell-derived proline enhances CAF collagen production. This crosstalk contributes to the enhanced invasiveness and fibrosis of cavernous sinus tumors, which is relevant to surgical challenges and potential therapeutic targeting—consistent with the recognized potential of targeting intercellular crosstalk for cancer therapy (41, 42).

Taken together, our findings indicate that the increased invasiveness of cavernous sinus tumors is associated with a tumor cell-CAF positive feedback loop—tumor cells generate proline via Warburg effect-mediated metabolic reprogramming, stimulating CAFs to produce collagen and secrete IGF/IGF1 to enhance tumor progression and fibrosis, which complicates surgical resection (the first-line treatment for invasive PitNETs); targeting this loop (e.g., IGF1R, proline metabolism) may improve surgical outcomes, which we will further explore for targeted therapy development.

## Ethics Approval and Consent to Participate

This study has been approved by the Ethics Committee of Zhongshan Hospital of Fudan University. Written informed consent was obtained from all participants or their legally authorized representatives prior to enrollment in the study.

## Consent for Publication

All authors have reviewed and approved the manuscript for submission to the journal.

## Data Availability

All Mass Spectrum raw data have been deposited to iProX (https://www.iprox.org/) and can be accessed with the iProX entry under dataset identifier IPX000768002 (https://www.iprox.cn/page/DSV021.html;?url=1772246271353fqk5; password: ay0D).

## Supplemental data

This article contains supplemental data.

## Conflict of interest

The authors declare that they have no conflicts of interest with the contents of this article.
