# Supplementary figures and images for: Region-Resolved Integrative Multi-Omic Characterization Reveals Diverse Tumor and Microenvironment Features of Pituitary Neuroendocrine Tumors
Source: Mol Cell Proteomics. 2026 May 12;25(6):101583. doi: 10.1016/j.mcpro.2026.101583 (PMC13279300; doi:10.1016/j.mcpro.2026.101583)

# Supplementary Figure 1

A

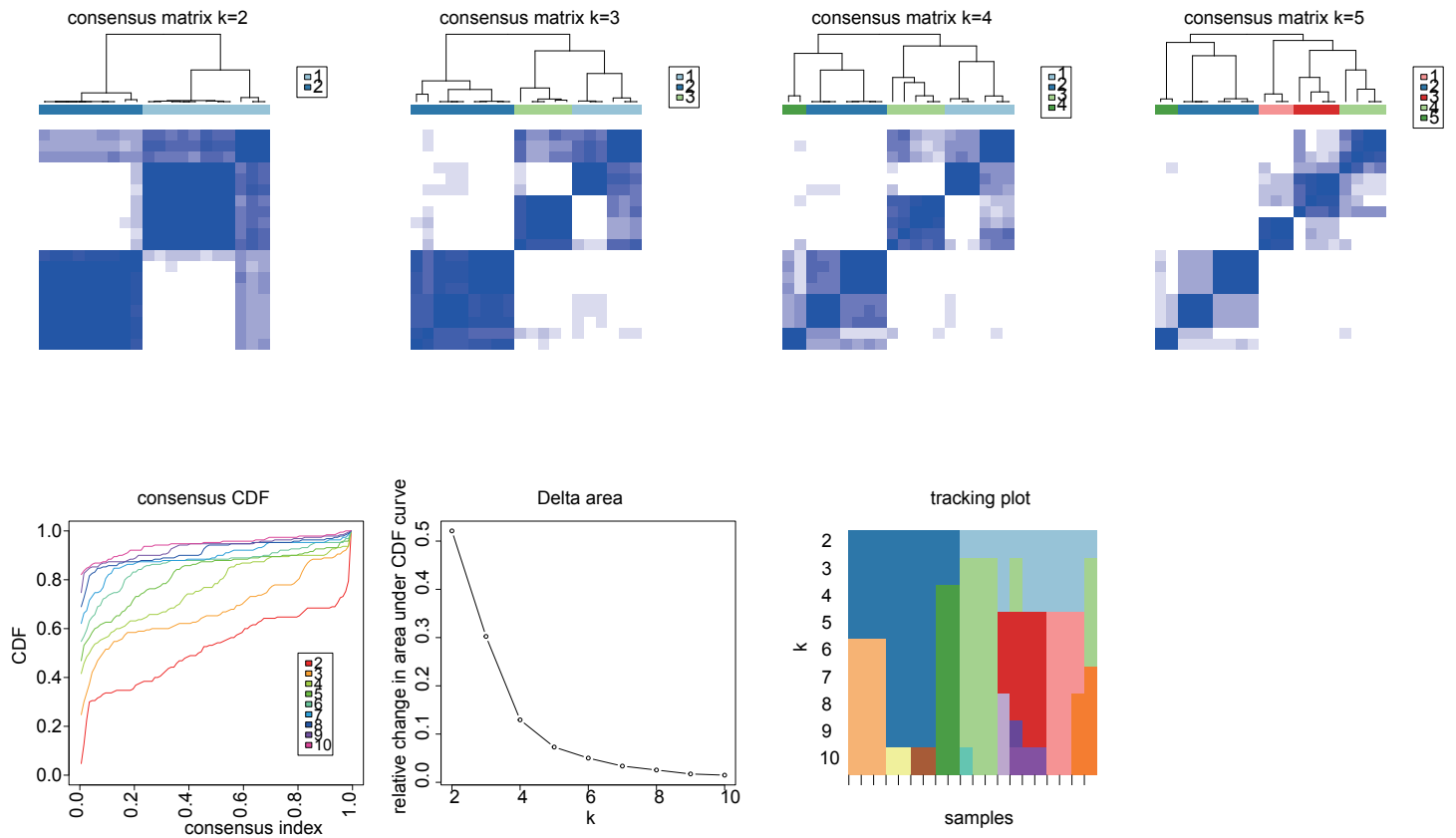

Supplement: Supplemental Figure 1 [file mmc1.pdf]

## Supplementary Figure 2

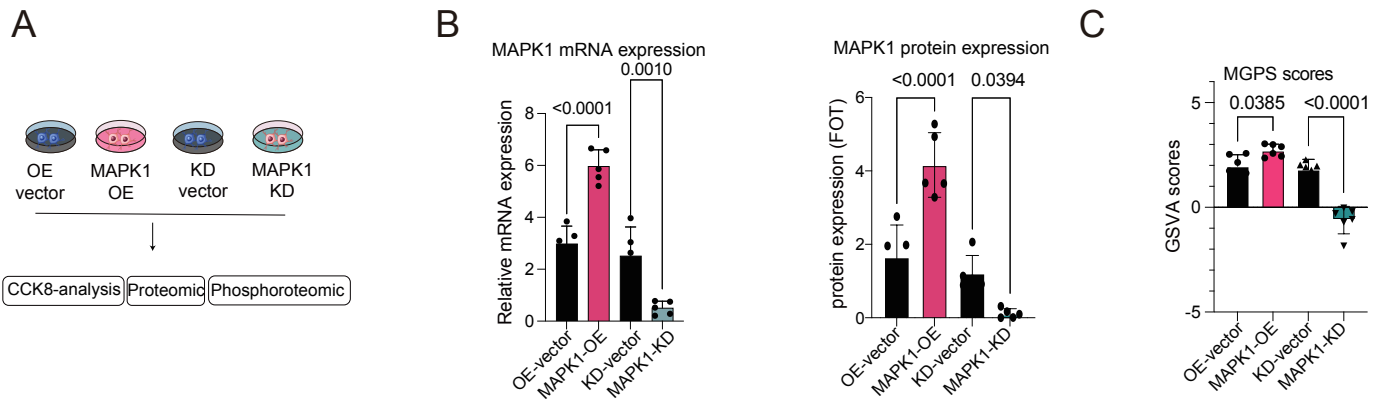

Supplement: Supplemental Figure 2 [file mmc2.pdf]
