# Supplementary material for: Region-Resolved Integrative Multi-Omic Characterization Reveals Diverse Tumor and Microenvironment Features of Pituitary Neuroendocrine Tumors
Source: Mol Cell Proteomics. 2026 May 12;25(6):101583. doi: 10.1016/j.mcpro.2026.101583 (PMC13279300; doi:10.1016/j.mcpro.2026.101583)
Supplement: Supplementary Table 1 [file mmc3.docx]

|  | **P1** | **P2** | **P3** | **P4** | **P5** | **P6** | **P7** | **P8** | **P9** | **P10** |
| --- | --- | --- | --- | --- | --- | --- | --- | --- | --- | --- |
| **Gender** | **F** | **F** | **F** | **F** | **M** | **M** | **M** | **M** | **F** | **F** |
| **Age** | **28** | **45** | **59** | **38** | **49** | **25** | **39** | **40** | **38** | **40** |
| **Knosp** | **4** | **4** | **4** | **4** | **4** | **4** | **4** | **4** | **4** | **4** |
| **PIT-1** | **-** | **+** | **-** | **+** | **+** | **-** | **-** | **+** | **+** | **-** |
| **T-PIT** | **+** | **-** | **+** | **+** | **+** | **-** | **-** | **-** | **-** | **+** |
| **SF-1** | **-** | **-** | **-** | **-** | **-** | **-** | **+** | **-** | **-** | **-** |
| **TSH** | **+** | **+** | **-** | **+** | **+** | **+** | **-** | **+** | **-** | **-** |
| **PRL** | **-** | **-** | **-** | **-** | **-** | **-** | **-** | **-** | **-** | **-** |
| **GH** | **-** | **+** | **-** | **+** | **+** | **-** | **-** | **-** | **+** | **-** |
| **ACTH** | **+** | **-** | **-** | **-** | **-** | **-** | **-** | **+** | **-** | **+** |
| **LH** | **-** | **-** | **-** | **-** | **-** | **-** | **+** | **-** | **-** | **-** |
| **FSH** | **-** | **-** | **-** | **-** | **-** | **-** | **-** | **-** | **-** | **-** |

Supplementary Table 1

The table summarizes, for ten patients, the baseline characteristics (sex, age, Knosp grade) and the immunohistochemical reactivity of the pituitary lineage-specific transcription factors PIT-1 (Pituitary Transcription Factor-1), T-PIT (T-box family transcription factor), and SF-1 (Steroidogenic Factor-1), together with the hormonal products TSH (Thyroid-Stimulating Hormone), PRL (Prolactin), GH (Growth Hormone), ACTH (Adrenocorticotropic Hormone), LH (Luteinizing Hormone), and FSH (Follicle-Stimulating Hormone).
